# Supplementary material for: Highly Sensitive Nanomagnetic Quantification of Extracellular Vesicles by Immunochromatographic Strips: A Tool for Liquid Biopsy
Source: Nanomaterials (Basel). 2022 May 6;12(9):1579. doi: 10.3390/nano12091579 (PMC9101557; doi:10.3390/nano12091579)
Supplement: Supplementary file 1 [file nanomaterials-12-01579-s001.zip › nanomaterials-1680883-supplementary.pdf]

Supplementary Materials

Highly Sensitive Nanomagnetic Quantification of Extracellular Vesicles by Immunochromatographic Strips: A Tool for Liquid Biopsy

Vera A. Bragina [1,†], Elena Khomyakova [1,†,‡], Alexey V. Orlov [1,2], Sergey L. Znoyko [1], Elizaveta N. Mochalova [1,3],
Liliia Paniushkina [4], Victoria O. Shender [5,6], Thalia Erbes [7], Evgeniy G. Evtushenko [8], Dmitry V. Bagrov [8],
Victoria N. Lavrenova [5,8], Irina Nazarenko [4,9] and Petr I. Nikitin [1,10,*]

[1] Prokhorov General Physics Institute of the Russian Academy of Sciences, 38 Vavilov St., 119991 Moscow, Russia; bragina_vera@nsc.gpi.ru (V.A.B.); elena.khomyakova@exosome-analytics.com (E.K.); alexey.orlov@kapella.gpi.ru (A.V.O.); znoykos@yandex.ru (S.L.Z.); mochalova@phystech.edu (E.N.M.)
[2] Moscow Institute of Physics and Technology, 9 Institutskii per., 141700 Dolgoprudny, Russia
[3] Sirius University of Science and Technology, 1 Olympic Ave., 354340 Sirius, Russia
[4] Institute for Infection Prevention and Hospital Epidemiology, Medical Center—University of Freiburg, Faculty of Medicine, University of Freiburg, 79106 Freiburg, Germany; paniushkina.liliia@gmail.com (L.P.); irina.nazarenko@uniklinik-freiburg.de (I.N.)
[5] Federal Research and Clinical Center of Physical-Chemical Medicine of the Federal Medical and Biological Agency, 1a Malaya Pirogovskaya St., 119992 Moscow, Russia; victoria.shender@gmail.com (V.O.S.); pkviktoria@mail.ru (V.N.L.)
[6] Shemyakin-Ovchinnikov Institute of Bioorganic Chemistry of the Russian Academy of Sciences, 16/10 Miklukho-Maklaya St., 117997 Moscow, Russia
[7] Department of Obstetrics and Gynecology, Medical Center—University of Freiburg, Faculty of Medicine, University of Freiburg, 79106 Freiburg, Germany; thalia.erbes@uniklinik-freiburg.de
[8] Lomonosov Moscow State University, 1 Leninskie Gory, 119991 Moscow, Russia; evtushenko@enzyme.chem.msu.ru (E.G.E.); bagrov@mail.bio.msu.ru (D.V.B.)
[9] German Cancer Consortium (DKTK), Partner Site Freiburg and German Cancer Research Center (DKFZ), 69120 Heidelberg, Germany
[10] National Research Nuclear University MEPhI (Moscow Engineering Physics Institute), 31 Kashirskoe Shosse, 115409 Moscow, Russia
* Correspondence: petr.nikitin@nsc.gpi.ru; Tel.: +7-(999)-910-20-69
† These authors contributed equally to this work.
‡ Current address: Exosome Analytics, 4 Rue Pierre Fontaine, 91058 Évry-Courcouronnes, France.

Citation: Bragina, V.A.; Khomyakova, E.; Orlov, A.V.; Znoyko, S.L.; Mochalova, E.N.; Paniushkina, L.; Shender, V.O.; Erbes, T.; Evtushenko, E.G.; Bagrov, D.V.; et al. Highly Sensitive Nanomagnetic Quantification of Extracellular Vesicles by Immunochromatographic Strips: A Tool for Liquid Biopsy. *Nanomaterials* **2022**, *12*, 1579. https://doi.org/10.3390/10.3390/nano12091579

Academic Editor: Ovidiu Crisan

Received: 29 March 2022
Accepted: 2 May 2022
Published: 6 May 2022

Publisher's Note: MDPI stays neutral with regard to jurisdictional claims in published maps and institutional affiliations.

Copyright: © 2022 by the authors. Licensee MDPI, Basel, Switzerland. This article is an open access article distributed under the terms and conditions of the Creative Commons Attribution (CC BY) license (https://creativecommons.org/licenses/by/4.0/).

S1. Isolation of Extracellular Vesicles

The conditioned media from MDA-MB-231 cells was first centrifuged at 500×g for 15 min, then at 10,000×g for 30 min at 4 °C in an FA-45-6-30 rotor (Eppendorf, Germany). The conditioned media from HT29 cells was centrifuged twice at 4,000×g for 20 min at 4 °C in the FA-45-6-30 rotor, concentrated with 100 kDa Amicon Ultra-15 Centrifugal Filter Units (Millipore, USA), and washed twice with PBS buffer.

For isolation of EVs from ascites samples of ovarian cancer patients, 1 mL of the ascitic fluid was centrifuged at 500×g for 15 min, then at 10,000×g for 30 min in an F-45–24-11 rotor (Eppendorf, Germany) at 4 °C. Then, 1 mL of PBS buffer was added to the supernatant, concentrated with a 1,000 kDa VivaSpin spin concentrator (Sartorius AG, Germany), and washed twice with PBS buffer using the same concentrator. An additional purification of ascites vesicles was performed via three-step sucrose gradient (48%, 40%, and 20% w/v) centrifugation in an MLS-50 rotor (Beckman Coulter, USA). The 100,000×g pellet was dissolved in 600 µL of 48% (w/v) sucrose. Then, 500 mL of 40% sucrose was overlaid on the first layer containing EVs. The third low-density layer was formed by 4 mL of 20% sucrose. The samples were centrifuged at 100,000 rpm for 3 h in the MLS-50 rotor at 4 °C. Prior to use, 62% sucrose was purified in the 100 kDa Amicon filters (Millipore, USA) to remove vesicle-like particle contamination of sucrose. The extracellular vesicles floated on 40% sucrose and those concentrated on the interface between the layers of 40% and 48% sucrose were collected, washed twice with PBS buffer, and concentrated using 100 kDa Amicon filters (Millipore, USA).

EVs derived from human serum samples of breast cancer patients and healthy donors were isolated using a qEV35 Size Exclusion Chromatography (SEC) column (IZON, UK). Briefly, the qEV35 (original) column was set in a vertical position and left at room temperature (RT) for 15 min. Then, 15 mL of sterile PBS was applied to the column. When the flow stopped, 0.4 mL of serum samples were loaded, and the void volume (3 mL, six first fractions) was immediately collected. When the sample completely ran into the column, and dropping stopped, 2 mL of PBS was added to keep the column running, and 0.5 mL fractions were collected after the void volume. Each of the fractions was characterized according to the Minimal Information for Studies of Extracellular Vesicles (MISEV) criteria. The particle quantity was assessed by Nanoparticles Tracking Analysis (NTA), while the presence of EV-characteristic biomarkers was measured using the enzyme-linked immunosorbent assay (ELISA).

S2. Nanoparticle Tracking Analysis

The size and concentration of EVs isolated from cell culture supernatants, as well as from ascites of ovarian cancer patients, were measured by NTA using a Nanosight LM10 HSBF instrument (Nanosight, UK). The instrument configuration, setups, and measuring procedure were the same as described in detail in [50]. Briefly, the configuration included a 405 nm, 65 mW laser unit with passive temperature readout and a highly sensitive EMCCD-type camera, using NTA 2.3 (build 33) software (Nanosight, UK). The samples were diluted by particle-free PBS to reach a concentration around 1.5×10^{8} particles/mL. Fourteen 60 s videos were recorded in the advanced mode (shutter = 850, gain = 450, lower threshold = 910, higher threshold = 11180). The processing was performed in basic mode (detection threshold = 9 multi, min expected size = 30 nm). The particles from all recorded videos were collected in a single table (5700–7400 total tracks) followed by calculation of the joined PSD histogram, mean size, and total particle concentration, corrected by the dilution factor used for each particular sample.

The particle quantity and size distribution of EV fractions purified from the human serum samples of breast cancer patients and healthy donors were determined by NTA using a PMX110 device (Particle Metrix, Germany). The samples were diluted 1:1000 in 0.1xPBS (pre-filtered with a 0.22 µm filter) to a final volume of 1 mL. The capturing settings were as follows: camera sensitivity = 85.0%, shutter speed range = 70, scattering intensity = 4.0, cell temperature = 25 °C. The videos were analyzed using ZetaView Software (8.04.02, Germany) at a maximum particle size = 1000 nm, a minimum particle size = 10 nm, and a minimum particle brightness = 20 AU (arbitrary units). The statistical analysis was performed using GraphPad Prism 8.1.0 (GraphPad Software, USA). Significance was calculated using the multiple comparisons *t*-test (Holm–Sidak method).

S3. Transmission Electron Microscopy (TEM)

HT29 EVs: carbon-coated TEM grids (Ted Pella, USA) were pre-treated for 45 s with a glow discharge device Emitech K100X (Quorum Technologies, UK) to make the surface hydrophilic for better EV adsorption. Then, 10 μL of HT29 EV suspension was deposited onto the grid for 2–3 min and stained with 1% uranyl acetate for 1–2 min. The EV images were obtained using a transmission electron microscope JEM-1400 (Jeol, Japan) operating at 120 kV. Overall, more than 45 images were quantified using the ScanEV online service [51].

MDA-MB-231 EVs: 10 μl of MDA-MB-231 EV suspension was loaded on a 300-mesh copper grid and fixed with 1 % glutaraldehyde. After washing with double-distilled water, the samples were negatively stained with 10 μl of 1% uranyl acetate. The images were taken using an electron microscope LEO 906E (Zeiss, Germany) using SIS software (Olympus, Germany).

S4. Analysis of EV markers with ELISA

The distribution of CD9 markers in the SEC fractions of EVs isolated from the serum of breast cancer patients was analyzed with ELISA kits (MyBioSource, USA), according to the manufacturer's guidelines. Briefly, the provided standards and 100 μL of each SEC fraction were applied on a microplate pre-coated with anti-CD9 antibodies and incubated at 37 °C for 2 h. After washing steps, a biotin-conjugated antibody provided with the kit was added. The number of bound antibodies was visualized using streptavidin-coupled horseradish peroxidase. The substrate was added and incubated at 37 °C for 20 min with light protection and the optical density was measured at 450 nm using an Infinite M Plex TECAN plate reader. The CD9 concentrations were determined using the standard curve equation.

S5. Characterization of EVs Isolated from the Serum of Breast Cancer Patients Using Size-Exclusion Chromatography

For seven SEC fractions of EVs, the particle quantities and CD9 contents were assessed by NTA and ELISA, respectively (Figure S1). The void fraction was used for the negative control. The fractions 2 and 3 showed the highest levels of EV markers. The respective particle quantities were used for the follow-up analysis.

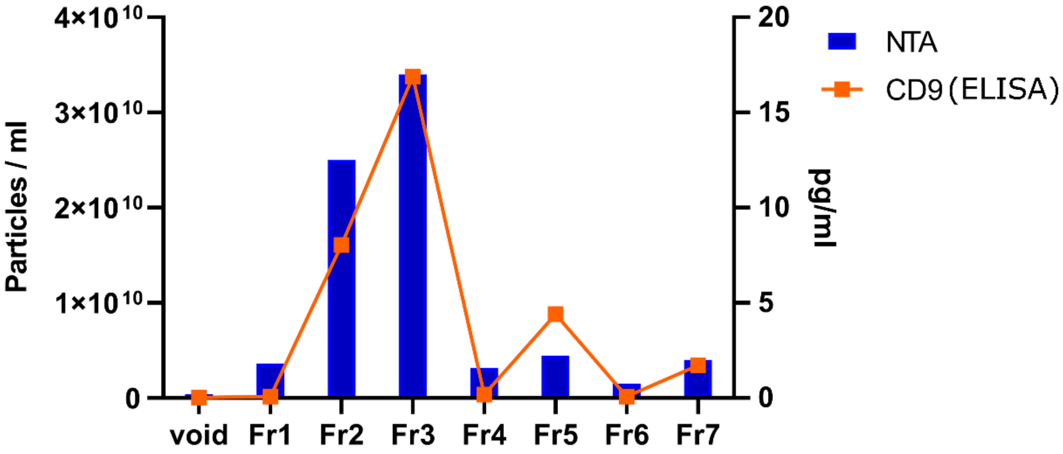

Figure S1. NTA and ELISA characterization of EVs derived from human serum samples of breast cancer patients.

S6. Flow Cytometry Analysis of Cells

The HT29 and MDA-MB-231 cells were analyzed by flow cytometry for expression of CD9, CD81, and EpCAM surface membrane proteins. The HT29 cells cultured in 225 cm^2 at 80-90% confluence were detached with a solution of PBS with ethylenediaminetetraacetic acid (EDTA; PanEco, Russia) and washed twice in PBS buffer. Then, 1×10^6 HT29 cells were incubated with 20 μL of PE-anti-CD9, PE-anti-CD81, or

PE-anti-EpCAM antibodies (cat. nos. 341648, 555676, and 347198, BD Biosciences, respectively) and the corresponding PE mouse IgG1 isotype control (cat. no. 555749, BD Biosciences) for 1 h at RT in the dark on a rotator. After staining, the HT29 samples were analyzed with an LSRFortessa™ Flow Cytometer (BD Biosciences, USA).

The expression of CD9, CD81, and EpCAM on the MDA-MB-231 cells was analyzed as previously described [52]. Briefly, 1×10^6 of MDA-MB-231 cells (approximately 90% confluence, from the well of a six-well plate) were detached by exposure to PBS-EDTA, washed three times with PBS buffer, and incubated with 20 μL of PE-anti-CD9, APC-anti-CD81, or PE-anti-EpCAM antibodies (cat. no. 341647, 551112, and 347198, BD Biosciences, respectively), and the corresponding PE mouse IgG1 and APC mouse IgG1 (cat. nos. 555749 and 555751, BD Biosciences, respectively) isotype controls. The reactions were carried out at RT in the dark in a rotator for 1 h (for anti-CD9 and anti-CD81 staining) or for 2 h (for anti-EpCAM staining). After the staining, the MDA-MB-231 samples were analyzed with a NovoCyte Flow Cytometer (ACEA Biosciences, USA).

S7. Analysis of Expression of Membrane Surface Proteins on HT29 and MDA-MB-231 Cells with Conventional Flow Cytometry

For accurate quantification of the total EV population, membrane surface proteins common to the majority of EVs are usually applied. To select appropriate membrane antigens, HT29 and MDA-MB-231 cells were analyzed for expression of CD9, CD81, and EpCAM surface proteins by conventional flow cytometry. In the obtained flow cytometry histograms for PE-Anti-CD9- and PE-Anti-CD81-stained HT29 cells, as well as for PE-Anti-CD9- and APC-Anti-CD81-stained MDA-MB-231 cells (Figure S2A–D), expression of CD9 and CD81 tetraspanins was observed on an absolute majority of both types of cells. The results indicate that the CD9 and CD81 protein markers are suitable for quantification of the total population of HT29 and MDA-MB-231 EVs.

We also studied expression of EpCAM on the HT29 and MDA-MB-231 cells. It was found (Figure S2E and F) that HT29 cells were totally positive for EpCAM with a very important difference in the fluorescence peak values between PE-Anti-EpCAM and PE-IgG1-isotype control staining. The fluorescence histograms of PE-Anti-EpCAM and PE-IgG1-stained MDA-MB-231 cells were slightly superposed. Nevertheless, the flow cytometry data confirmed that the EpCAM-positive cells represented the major population (95%) of MDA-MB-231 cells. The lower mean value of PE fluorescence for EpCAM in the case of MDA-MB-231 cells could be explained by a smaller quantity of EpCAM antigens on each MDA-MB-231 cell compared to the HT29 cells. Thus, the CD9, CD81, and EpCAM membrane surface proteins were found to be good experimental models as targets for EV quantification with the proposed IC tool.

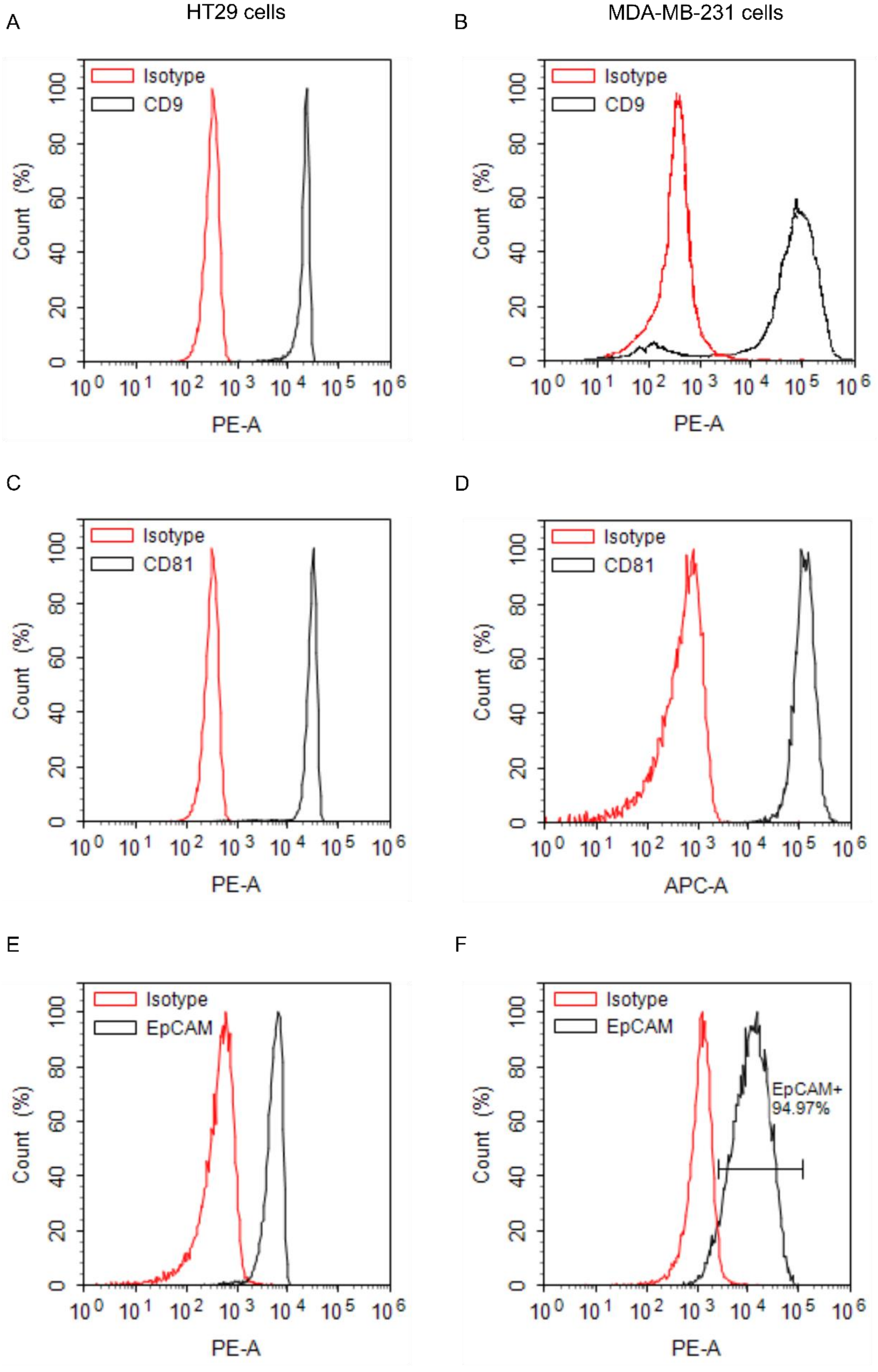

Figure S2. Analysis of expression of membrane surface proteins on HT29 and MDA-MB-231 cells with conventional flow cytometry: (A, B) CD9; (C, D) CD81; (E, F) EpCAM.

S8. Gating Strategy during Characterization of the EV–anti-CD9-MP Complexes with Imaging Flow Cytometry

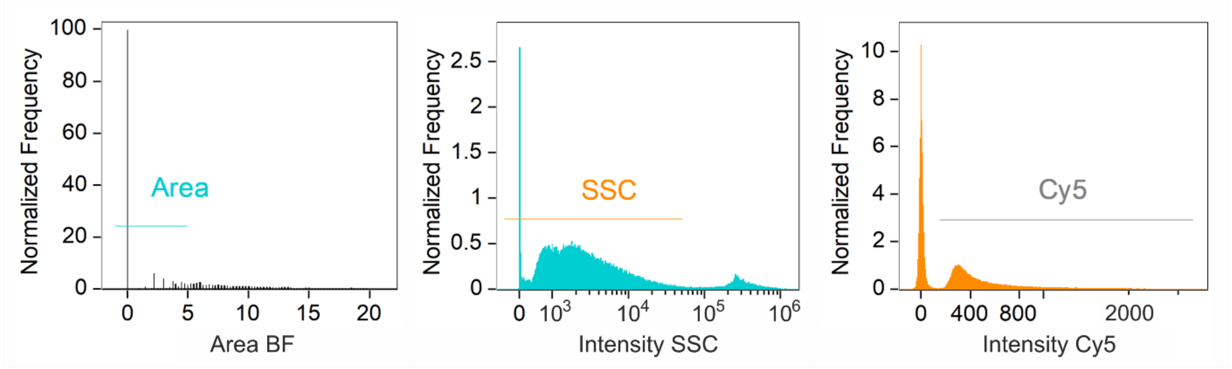

Figure S3. Gating strategy.

S9. Visualization of Formation of "EV–antibody-functionalized MP" Immune Complexes by Imaging Flow Cytometry

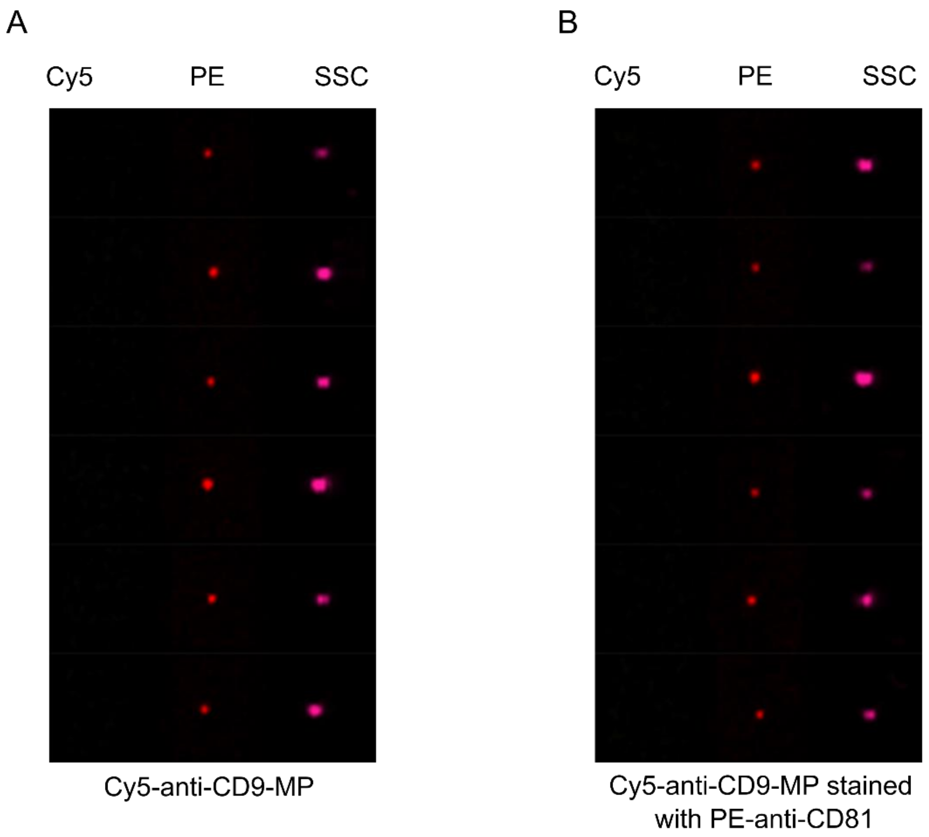

Figure S4. Images of control samples containing (A) Cy5-Anti-CD9-MP and (B) Cy5-Anti-CD9-MP with PE-Anti-CD81 in Cy5, PE, and side scatter (SSC) channels.

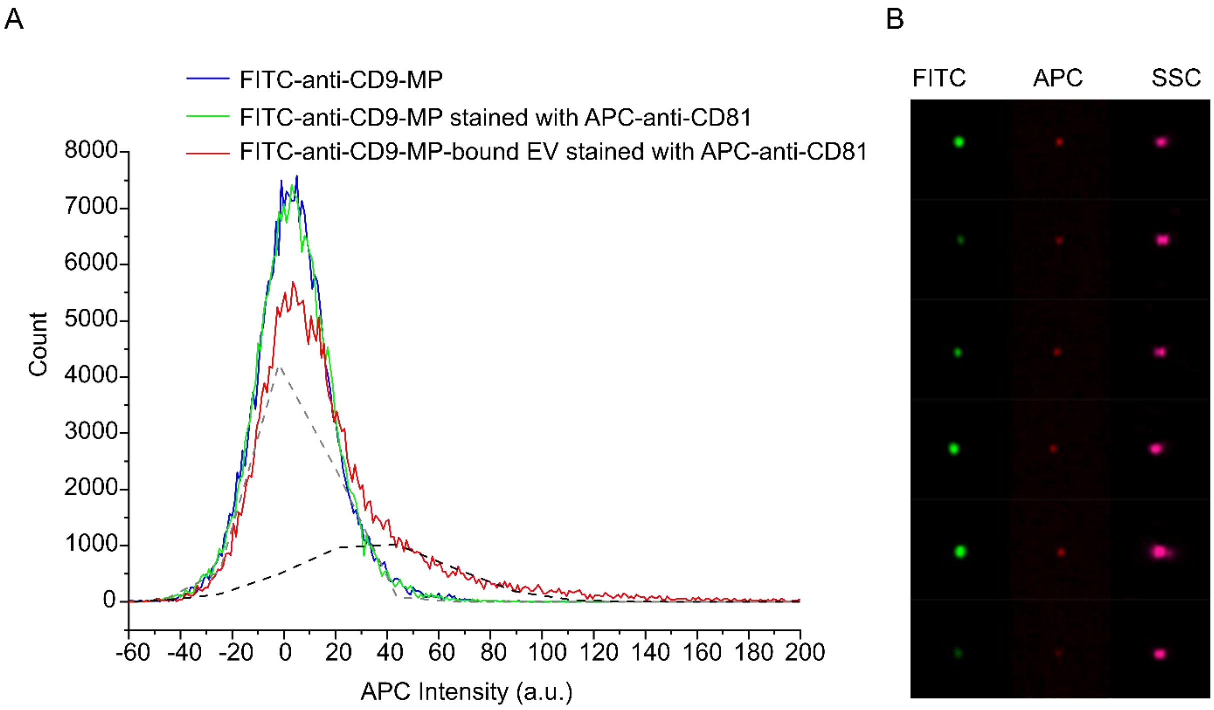

Figure S5. Analysis of binding of APC-anti-CD81-stained HT29 EVs to FITC-anti-CD9-MP with imaging flow cytometry: (A) APC intensity histograms of the analyzed samples: (1) FITC-anti-CD9-MP (blue line); (2) FITC-anti-CD9-MP stained with APC-anti-CD81 (green line); and (3) FITC-anti-CD9-MP-bound EV stained with APC-anti-CD81 (red line). The histogram for the buffer-only sample is not shown because almost all events from this control sample have been excluded as a result of gating. Fitting with two Gaussian distributions of the histogram of the FITC-Anti-CD9-MP-bound EVs stained with APC-Anti-CD81 are shown by dashed lines (grey—unbound FITC-Anti-CD9-MP; black—EV–FITC-Anti-CD9-MP immune complexes). Thirty percent of the population of FITC-positive objects are APC-positive. (B) Representative images of the formed immune complexes between FITC-Anti-CD9-MP and APC-anti-CD81-labelled EVs in FITC, APC, and side scatter (SSC) channels.

S10. Calibration Plots for EpCAM^{+}/CD9^{+} HT29 and MDA-MB-231 EVs

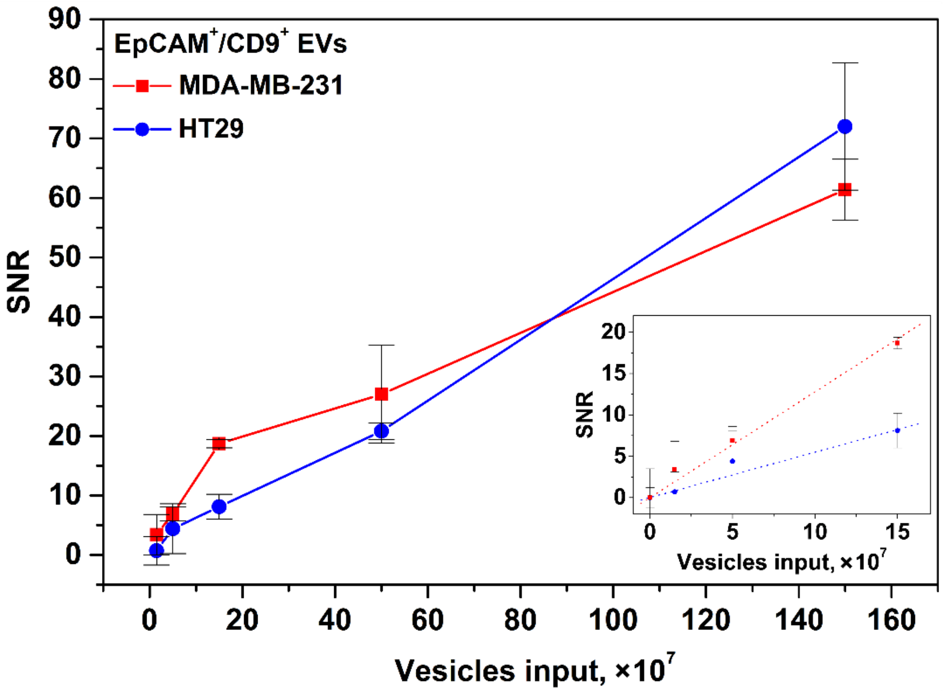

Figure S6. Calibration plots for EpCAM+/CD9+ HT29 and MDA-MB-231 EVs. Insert: Zoomed linear fitting plots for the low EV input range.

The detection limits for EpCAM+/CD9+ HT29 and MDA-MB-231 EVs (Figure S6) are $3.6x10^7$ and $1.6x10^7$ particles/test ($12x10^5$ and $5.3x10^5$ EV/μL), respectively.

References

50. Evtushenko, E.G.; Bagrov, D.V.; Lazarev, V.N.; Livshits, M.A.; Khomyakova, E. Adsorption of extracellular vesicles onto the tube walls during storage in solution. *PLoS One* **2020**, *15*, e0243738, doi:10.1371/journal.pone.0243738

51. Nikishin, I.; Dulimov, R.; Skryabin, G.; Galetsky, S.; Tchevkina, E.; Bagrov, D. ScanEV-A neural network-based tool for the automated detection of extracellular vesicles in TEM images. *Micron* **2021**, *145*, 103044, doi:10.1016/j.micron.2021.103044

52. Anufrieva, K.S.; Shender, V.O.; Arapidi, G.P.; Pavlyukov, M.S.; Shakhparonov, M.I.; Shnaider, P.V; Butenko, I.O.; Lagarkova, M.A.; Govorun, V.M. Therapy-induced stress response is associated with downregulation of pre-mRNA splicing in cancer cells. *Genome Med.* **2018**, *10*, 1–18, doi:10.1186/s13073-018-0557-y
